# Effective Information Provision About the Side Effects of Treatment for Malignant Lymphoma: Protocol of a Randomized Controlled Trial Using Video Vignettes

**DOI:** 10.2196/12453

**Published:** 2019-05-02

**Authors:** Nanon Labrie, Sandra van Dulmen, Marie José Kersten, Hanneke JCM de Haes, Arwen H Pieterse, Julia CM van Weert, Dick Johan van Spronsen, Ellen MA Smets, INSTRUCT Group

**Affiliations:** 1 Medical Psychology Amsterdam Public Health Research Institute, Cancer Center Amsterdam Amsterdam University Medical Centers Amsterdam Netherlands; 2 Athena Institute Vrije Universiteit van Amsterdam Amsterdam Netherlands; 3 Netherlands Institute for Health Services Research (NIVEL) Utrecht Netherlands; 4 Radboud Institute for Health Sciences, Department of Primary and Community Care Radboud University Medical Center Nijmegen Netherlands; 5 Faculty of Health and Social Sciences University of South-Eastern Norway Drammen Norway; 6 Department of Hematology Cancer Center Amsterdam, Amsterdam University Medical Centers University of Amsterdam Amsterdam Netherlands; 7 Medical Decision Making Department of Biomedical Data Sciences Leiden University Medical Center Leiden Netherlands; 8 Amsterdam School of Communication Research University of Amsterdam Amsterdam Netherlands; 9 Department of Hematology Radboud University Medical Center Nijmegen Netherlands

**Keywords:** physician patient relationship, health communication, information dissemination, immediate recall, trust, symptoms, clinical trial protocol, video vignettes

## Abstract

**Background:**

Informing patients with cancer about the possible implications of prospective treatment is a crucial yet challenging task. Unfortunately, patients’ recall of medical information is generally poor and their information needs are not met. Effective information giving entails that oncologists help patients understand and recall the implications of their treatment, meanwhile fostering a trusting physician-patient relationship. Communication strategies that are often suggested to be effective are *structuring* and *tailoring* (cognition-oriented) but also are oncologists’ expressions of *caring* or empathy (affect-oriented).

**Objective:**

The aim of this study is to provide evidence concerning the pathways linking physician communication to (improved) consultation outcomes for patients. More specifically, the aim is to determine the effects of information *structuring* and information *tailoring*, combined with physician *caring*, on information recall, satisfaction with information, and trust in the physician (primary objective) and on symptom distress (secondary objective).

**Methods:**

A randomized controlled trial, systematically testing the effects of information *structuring* and information *tailoring*, each combined with *caring*, in 2 video-vignette experiments (2×2 and 2×2×2 design). Using an online survey platform, participants will be randomly allocated (blinded) to 1 of 12 conditions in which they are asked to view a video vignette (intervention) in which an oncologist discusses a treatment plan for malignant lymphoma with a patient. The independent variables of interest are systematically varied across conditions. The outcome measures are assessed in a survey, using validated instruments. Study participants are (former) patients with cancer and their relatives recruited via online panels and patient organizations. This protocol discusses the trial design, including the video-vignette design, intervention pretesting, and a pilot study.

**Results:**

Data collection has now been completed, and preliminary analyses will be available in Spring 2019. A total of 470 participants completed the first part of the survey and were randomized to receive the intervention.

**Conclusions:**

The results of the proposed trial will provide evidence concerning the pathways linking physician information, giving skills to (improved) consultation outcomes for patients.

**Trial Registration:**

Netherlands Trial Register NTR6153; https://www.trialregister.nl/trial/6022 (Archived by Webcite at http://www.webcitation.org/76xVV9xC8).

**International Registered Report Identifier (IRRID):**

DERR1-10.2196/12453

## Introduction

### Background

Informing patients with cancer about the possible implications of prospective treatment is a crucial yet challenging task. Cancer treatment plans are typically complex, and the effects on patients’ physical and psychological well-being can be severe. Although it is important that patients remember treatment information, research consistently shows that patients’ recall of medical information is poor [1-7]. Patients forget approximately 40% to 80% of the information that is provided by their oncologist [5,8-10].

Lack of information is not only potentially harmful but has also been cited as among the greatest causes of dissatisfaction in patients with cancer [1,6,11-13]. Patients mostly want information about treatment [14], particularly about symptoms and side effects, both in the short and long term [15-18]. Having information about symptoms and treatment may provide patients with a sense of control, reduce their anxiety and distress, and provide support coping with the physical and psychological demands of cancer treatment [18,19]. Additionally, by discussing current and future symptom experiences, physicians can influence patients’ expectations of symptoms and their ability to control symptoms [20]. These expectations may subsequently affect patients’ actual symptom experiences, either positively (placebo effect) or negatively (nocebo effect) [20,21].

Finally, providing comprehensive and understandable information that is congruent with patients’ needs is known to increase patients’ trust in the physician [22], which is associated with a higher tolerance for symptoms [21,23]. Indeed, patients with cancer who feel more able to cope with the disease and its treatment are better adjusted and experience greater quality of life than patients who feel less in control [24]. Therefore, effective information giving entails that oncologists help patients understand and recall the implications of their treatment, meanwhile fostering a trusting physician-patient relationship.

### Strategies of Effective Information Giving

Communication strategies to enhance information provision can be described as either cognition- or affect-oriented [25,26]. Cognition-oriented strategies are typically aimed at enhancing patient-related outcomes that are cognitive in nature, such as patients’ recall of information [27-31]. Two prominent cognition-oriented strategies are information *structuring* and *tailoring*. Affect-oriented strategies target patients’ emotions and include, for example, oncologists’ expressions of *caring* or empathy [9,31-35]. Owing to the inherent interplay between the cognition-oriented and affect-oriented aspects of information giving [36], these strategies should ideally be studied in conjunction.

#### Structuring

Structuring treatment information, that is, a clear organization of information provision during a consultation, is assumed to improve patients’ recall. Structure allows patients to systematically organize and store information in their working memory, such that it is easier to remember at a later moment [28,29]. Similar to the way in which newspaper articles or books are structured by means of, for example, (sub)titles and paragraph/chapter headings, physicians can use verbal structure *signals* to guide their patients through the information (also called the *book metaphor*) [28]. A total of 4 types of explicit verbal structure signals can be distinguished [37,38]: (1) Statements that set the agenda and announce key topics that will be dealt with in detail later (eg, “The most important issues to be discussed are...”); (2) Statements used to conclude or summarize the most important issues discussed (eg, “All in all, there are four main issues to be considered...”); (3) Ordinal or numeral signals that indicate elements of a series (eg, “first, second,...” and “in addition”); and (4) Statements expressing an opinion or a point of view (*“unfortunately”*; *“in my opinion”*) [37,38]. As early as in the 1970s, Ley et al found that the use of explicit structure signals in medical information can improve students’ memory [12]. Four decades later, Langewitz et al [28] demonstrated that providing verbal structure signals during discharge consults in emergency medicine significantly improved students’ recall. These results are yet to be replicated in clinical contexts.

#### Tailoring

Tailoring, that is, adjusting the (amount of) information to meet an individual patient’s information need, is proposed to be more effective than the provision of generic information [39,40]. People tend to pay more attention to information that they perceive as personally relevant, which leads to improved information processing and consequently better recall [40-42]. For patients with cancer, providing them with *less* information than they wish is known to cause dissatisfaction [16] and providing them with *more* information than they desire can also be harmful [43]. Consequently, a tailored approach, that is, congruence between the patients’ information need and physician's information provision, is generally advocated [40,41,43-46].

#### Caring

Caring refers to a communication style in which the physician displays behaviors of empathy for and affective engagement with the patient [9,47,48,49], thereby potentially reducing patients’ emotional distress [9,32,48] and enhancing patients’ memory of information [49,50]. A sense of a caring relationship with the physician has been shown to increase patients’ satisfaction with the provided information [34]. Other studies demonstrate that patients’ trust in their physician reduces the need to subsequently seek detailed information [44,51]. Moreover, research suggests that physicians can help alleviate symptom distress by affective communication, rather than by information giving alone [21,52].

The effectiveness of the aforementioned cognitive (ie, structuring and tailoring) and affective (ie, caring) strategies may differ, depending on patients’ individual characteristics known to affect information processing, such as their age [5], their degree of anxiety [35] or coping style [53], or their medical history.

### Research Objectives

The randomized controlled trial described in this study protocol aims to provide evidence concerning the pathways linking physician communication to (improved) consultation outcomes for patients. More specifically, it seeks to determine the effects of information *structuring* (experiment 1) and information *tailoring* (experiment 2), combined with physician *caring*, on information recall, satisfaction with information, and trust in the physician (primary objective). Additionally, it aims to determine the effects of these independent variables on expected symptom distress (secondary objective). The planned trial consists of a single study in which 2—analytically distinct—subexperiments can be identified—each with specific objectives related to the independent variables.

### Hypotheses and Research Questions

**Figure 1:**
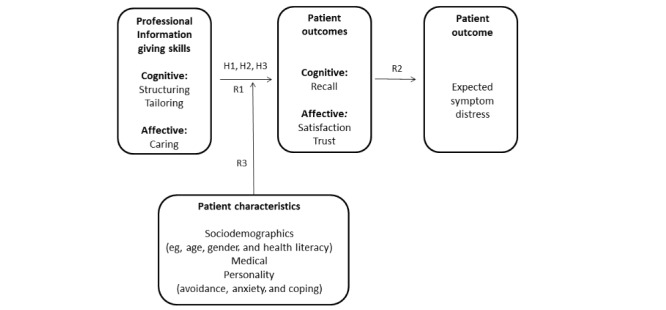
Hypotheses and research questions.

The hypotheses and research questions are as follows (see Figure 1):

1. Experiment 1: Structuring
  - H1: Information *structuring* positively affects patients’ recall of treatment information.
2. Experiment 2: Tailoring
  - H2: Information *tailoring* positively affects patients’ recall of treatment information.
3. Experiments 1 and 2: Caring
  - H3: Oncologists’ expressions of *caring* positively affect patients’ recall of, and satisfaction with, treatment information and their trust in the oncologist.
  - RQ1: Is there an interaction effect of information *structuring* or *tailoring* and oncologists’ expressions of *caring*?
  - RQ2: Do patients’ recall of, and satisfaction with, treatment information and their trust in the oncologist affect patients’ expected symptom distress?
  - RQ3: Do patient characteristics, including sociodemographics (eg, gender, age, and health literacy), medical history (eg, type and year of diagnosis and treatment), and personality traits (eg, coping style and trait anxiety), moderate the hypothesized relationships?

## Methods

### Trial Design

The trial employs a between-subjects single-message factorial design in which the independent variables of interest are systematically manipulated. This is done using the video vignette methodology. In experiment 1, hematologists’ information structuring and expressions of caring are varied in a 2 (standard versus enhanced structuring) × 2 (standard versus enhanced caring) design. In experiment 2, hematologists’ information tailoring and expressions of caring are varied in a 2 (high versus low need for information) × 2 (high versus low amount of information provided) × 2 (standard versus enhanced caring) design. This results in 12 experimental conditions, or interventions, across the trial (see Tables 1 and 2).

**Table 1:** Experiment 1: Manipulation of provider information structuring and caring (2×2 design).

Manipulations	Standard of care	Caring
Standard of care	Standard of care	Enhanced caring
Structuring	Enhanced structuring	Enhanced caring and structuring

**Table 2:** Experiment 2: Manipulation of provider caring and information tailoring in a match/mismatch design (2×2×2 design).

Manipulations		Provider			
		Enhanced caring		No caring	
		Additional information provision	No additional information provision	Additional information provision	No additional information provision
**Patient**					
	High information need	Tailoring match; (++^a^)	Tailoring mismatch; (+ −^b^)	Tailoring match; (++)	Tailoring mismatch; (+ −)
	Low information need	Tailoring mismatch; (− +^c^)	Tailoring match; (− −^d^)	Tailoring mismatch; (− +)	Tailoring match; (− −)

^a^Patient with high information need receiving additional information provision.

^b^Patient with high information need yet not receiving additional information.

^c^Patient with low information need nevertheless receiving additional information.

^d^Patient with low information need not receiving additional information.

### Study Setting

The trial is set within the specific context of hematology and the treatment of malignant lymphoma (diffuse large B-cell lymphoma [DLBCL]). Treatment information, including information about likely symptoms, is particularly important in this context because treatments are highly unpleasant and require patients’ commitment and adherence in the face of these demands [36]. The trial is conducted in the Netherlands. This study has been evaluated by the medical ethical committee of the Academic Medical Center. The institutional ethics committee has determined that the study is exempt from the need for review according to the Dutch regulations for research involving human subjects (W16_054 # 16.069, date: February 2016).

### Eligibility

Participants are (former) patients with cancer and their relatives. Eligible participants (1) have experience with oncology consultations, as a (former) patient or relative; (2) are fluent in Dutch; (3) are at least 18 years of age, and (4) have access to the internet at their home computer to complete the survey and view a video vignette. Inclusion of (former) patients with cancer and their relatives is believed to maximize identification with the patient displayed in the video vignette intervention.

### Video Vignette Intervention

#### Video Vignettes

Eligible participants are randomized in equal proportions to view 1 of 12 video vignette interventions. Video vignettes are scripted, hypothetical scenarios of real-life (medical) consultations, which allow for the systematic variation of verbal and/or nonverbal behaviors across experimental conditions [48,54-63]. Video vignettes are preferred in health communication research when ethical or practical considerations prevent the manipulation of physician behaviors in clinical practice. Participants are asked to view and evaluate vignettes while imagining themselves to be the patient in the video, that is, participants act as *analogue patients* [61,62]. Several studies demonstrated the validity of this type of methodology [60-63] and show that video vignettes allow for good levels of experimental control through script standardization and manipulation checks. Formal guidelines for video vignette development proposed by Hillen et al [61] and van Vliet et al [62] are used in this study to enhance internal and external validity.

#### Scenario Development

The first author (NL) used existing recordings (n=12) [64] and 2 days of real-life observations of consultations between hemato-oncologists and lymphoma patients to develop a script of a prototypical treatment-related consultation in hematology. Additionally, instructional materials for patients and evidence-based publications about malignant lymphoma and, in particular, DLBCL, its treatment (R-CHOP), and possible side effects were consulted. The main side effects and complaints associated with DLBCL and its treatment, as derived from the literature, included fatigue, nausea, infections, and anxiety.

When possible, exact excerpts from the transcribed consultations and information materials were embedded in the basic script to enhance ecological validity. To further ensure realism of the script, the basic script was discussed and revised in several discussion rounds with the project’s lead hematologist (MJK). Furthermore, the script was role played by 2 medical communication researchers to test the natural flow of the dialogue. The basic script was then sent out for commentary to an expert panel consisting of 8 hematologists, radiotherapists, and oncologists from academic and regional hospitals, ranging in experience from resident in training to senior attending. Additionally, 11 patients with a history of lymphoma or blood cancer were recruited to provide written feedback. This was done via PanelCom [65], an online panel for patient-provider research. Expert panel members were subsequently excluded from further trial participation. Finally, a professional scriptwriter also commented on the script. The physicians, patients, and scriptwriter were asked to specifically provide feedback on the script’s (medical) realism and the interaction between the physician and patient. The script was revised accordingly and discussed for final revision by the overall project group, including 4 medical psychologists, 1 hematologist, 3 medical communication researchers, and 2 medical education experts.

#### Experimental Manipulations

The basic script was designed such that the independent variables of interest—structuring, tailoring, and caring—could be integrated into the dialogue in the form of blocks of text fragments. These fragments sometimes consisted of a turn-taking sequence between the hematologist and patient or of short utterances added to the hematologist’s text. Experimental manipulations were thus operationalized primarily as verbal expressions, which were in turn supported by nonverbal behaviors, if possible (eg, using hand signals to support statements such as “first,” “second”).

##### Structuring

On the basis of theoretical conceptualizations of text structuring [37,38,66,67], providing structure when giving information was operationalized (1) by having the physician provide verbal signals that introduce content and set the agenda (eg, “In today’s consultation I will tell you more about the treatment with chemotherapy and what to expect”; “I would like to discuss four possible side-effects and complaints with you”), (2) by having the physician summarize information (eg, “In short, you could thus suffer from nausea, fatigue, and infections”), and (3) by having the physician use ordinal or numeral text signals to indicate separate elements in a series (eg, “first, second”; “additionally, moreover, finally”). These structure markers were absent in the standard script (see Table 2).

##### Tailoring

Patients’ need for information was operationalized in the video script by having the patient respond to a prompt by the physician (eg, “Would you like to know more about this?”). The patient either confirmed a preference for more information (eg, “I would like to know as much as possible”) or stated that the information received was considered sufficient for the time being (eg, “It’s clear for now. I would like to let it all sink in”). This was done twice, once at the beginning and once toward the end of the consultation, keeping the patient’s need for information (high vs low) consistent across the script. Tailoring was defined as a match between a high information need of the patient and the provision of further information (*tailoring*^*+*^) or a match between a low-information need of the patient and the absence of further information provision (*tailoring*^*−*^). In contrast, lack of tailoring was defined as a mismatch between need for information and information provision (*no tailoring*^*+*^ and *no tailoring*^*−*^; see Table 2).

##### Caring

Physician caring was operationalized based on Hillen et al [33] who developed, tested, and used doctors’ verbal expressions of caring in a scripted video vignette study to test the effect of caring on trust. These verbal utterances were modified to fit the hemato-oncology setting, based on feedback from the expert panel, the patients, screenwriter, and project group during script development. The overall effectiveness of these manipulations of provider caring was established previously [33]. In the standard script, these expressions of caring were absent (Table 2).

##### Pretest Script Manipulations

To test the efficacy of the manipulations pertaining to information structuring and information preference tailoring, a pretest was conducted among a convenience sample of 63 participants (76%, [48/63] female; age range: 20 to 72 years; mean 41.5), including 19% (12/63) physicians (5 of which were hematologists), 11% (7/63) patients with lymphoma and blood cancer, 33% (21/63) researchers, and 37% (23/63) participants without previous experience in hemato-oncology. Participants were randomly assigned to one of the experimental conditions in which they were asked to read short, relevant excerpts of the script. Depending on the condition, participants were asked to rate the extent to which the physician *structured* the provided information or *adjusted* the amount of information to the patient’s personal needs on a scale from 1 (not at all) to 10 (a lot). In an open-ended question, they were asked to explain their judgment. The findings from the pretest suggested that these manipulations were largely recognized. Nonetheless, participants did not always correctly distinguish between structured and unstructured information provision. To resolve this, information structuring signals were made more explicit in the script, for example, by reformulating text fragments more strongly and by emphasizing verbal statements with nonverbal behaviors.

#### Filming and Editing

The roles of physician and patient were played by professional actors with ample experience as standardized patients in the medical context and with video vignette research in particular. The role of the hematologist was played by a 51-year-old white male; the role of the patient was played by a 57-year-old white female. The video vignettes were recorded by a professional film crew, over the course of 2 days, at our hospital. The first day of filming was used as a training day and resulted in a preliminary video clip, shot with a single-camera setup. This clip was shown to a group of 10 medical communication and education experts who provided feedback on, for example, aspects such as quality of the image, as well as the acting skills and realism of the set. Changes were made where necessary. On the second day of filming, the entire script was filmed using a multicamera setup: the scenario was shot from 3 different angles. Subsequent editing resulted in 12 experimental video vignettes, ranging in length between 9 and 11 min.

### Outcomes, Survey Development, and Testing

#### Survey (Outcome) Measures

As preparatory work for the trial, the experimental survey, including the study outcome measures, was developed and tested in a pilot study.

##### Background Measures

Survey questions concerning participants’ sociodemographic background included gender, birth year, ethnicity, living situation, educational level, and occupational status. In addition, questions concerning participants’ health literacy [68], medical knowledge (general and lymphoma-specific), overall health (1 item), and cancer (treatment and family) history were included. Personality trait measures included the avoidance scale of the Impact of Event Scale (8 items, 4-point scale) [69] to assess the tendency to avoid cancer-related issues, the Trait Anxiety Inventory (20 items, 4-point scale) [70] for the assessment of generalized anxiety, the Threatening Medical Situations Inventory (TMSI-2, Monitoring scale) [71] to assess a monitoring coping style, and a single item assessing information preferences in medical consultations (5-point scale).

##### Manipulation Checks

To assess manipulation success of the independent variables, 3 items similar to those used in the pretest were included (scale 1-10; perceived structuring, tailoring, and caring). Open text boxes were added for participants to explain their judgments. The Video Engagement Scale (15 items; 7-point scale) [60] was included to measure participants’ involvement with the video vignette.

##### Information Recall

Information recall was measured following the protocol of the Netherlands Patient Information Recall Questionnaire [8]. On the basis of the video vignette script, an item pool was developed, pairing open-ended questions (active recall) with analogous multiple-choice questions (recognition). A code sheet was developed by the authors (NL and ES) to assess correctly recalled items and calculate active recall and recognition scores. The results from the pilot test were used to refine the scale items. Two coders independently scored the pilot answers. In the case of disagreement, results were discussed to reach consensus (for further details, see pilot testing results below).

Participants’ *satisfaction with the information* provided in the video was measured with 7 single items (5-point Likert scale). In total, 4 items were taken from the European Organisation for Research and Treatment of Cancer Quality of Life Questionnaire-INFO25 survey (items 52-55) [72]. Items assessed participants’ satisfaction with the *content* and *amount* of information provided by the hematologists; their *desire for more* or *less* information; the perceived *usefulness* and *clarity* of the information; and their satisfaction with the hematologist’s *information giving style*. Participants were asked to explain their answer in an open-ended question box.

To assess participants’ *trust* in the video hematologist, the 5-item short trust in oncologist scale (5-point Likert scale) was added [73].

##### Expected Symptom Distress

*Expected symptom distress,* that is, the perceived probability (1=very improbable to 5=very probable), severity (1=not at all severe to 5=very severe), and controllability (1=very little to 5 a lot) of physical as well as emotional distress was measured using separate items for each of the possible complaints discussed by the hematologist (fatigue, nausea, infections, and anxiety). Hemato-oncologists whom we consulted for this study differed in their opinion as to whether patients would appreciate information about the possible evolution of anxiety. For this reason, we added a single item (1=very unimportant to 5=very important) to measure the extent to which participants find it important that hematologists explicitly discuss possible feelings of anxiety and insecurity following diagnosis and treatment.

#### Pilot Testing

Survey validity and usability as well as the ecological validity of the video vignettes were pilot tested among 53 healthy participants aged 45 years and above. This age range was based on the incidence of non-Hodgkin lymphoma, which occurs mostly in older adults [74]. Participants were recruited in collaboration with Qualtrics panel services. In total, 500 invitation emails were sent out through the panel. A total of 145 (29.0%, 145/500) participants entered the survey, 87 (60.0%, 87/145) of which were filtered out as they did not match our participant requirements (Dutch; aged 45 years and older; and equal distribution of gender and region). Of the remaining 58 participants, a total of 53 (91%, 53/58) completed the survey including 1 random version of the 12 video vignettes.

The majority of participants (98%, 52/53) were Dutch. Participants (51%, 27/53 male) were on average aged 55.2 years (range 41 to 71); 64% (34/53) of participants indicated to have a partner. Participants had a diverse educational background; 42% (24/53) had a high school diploma; 30% (1/53) had completed vocational training; and 26% (14/53) had obtained a higher educational degree. In total, 45% (24/53) of the participants were not employed at the time of the survey. This was likely because of the average age of the target population.

Participants indicated to have little to average (medical) knowledge about cancer and lymphoma in particular (*cancer:* mean 2.68, range 1 to 5; *lymphoma*: mean 1.94, range 1 to 5). They judged their own health as average (mean 2.68; range 1 to 4) as compared with others their age. The majority of participants (79%) knew someone in their direct circle of friends and family who has (had) cancer. In total, 6 participants (11%) had received a cancer diagnosis (between 1993 and 2015), including skin cancer (4), breast cancer (1), and vocal cord cancer (1); two had received chemotherapy treatment.

Participants found the video realistic (mean 5.81; range 1 to 7), believable (mean 6.04; range 1 to 7), and the events displayed lifelike (mean 6.15; range 2 to 7). They found it easy to pay attention to the video (mean 5.85; range 1 to 7). More so, they perceived the physician as friendly (mean 5.96; range 1 to 7), likeable (mean 5.87; range 1 to 7), and credible in both his behavior (mean 5.94, range 3 to 7) and looks (mean 6.06, range 4 to 7). This provided support for the ecological validity of the video vignettes.

On average, participants found the physician’s information structured (mean 8.13, range 3 to 10). They also deemed the amount of information provided by the physician quite adapted to the patient’s needs (mean 7.72, range 2 to 10). Finally, participants perceived the physician as relatively empathetic (mean 7.57, range 3 to 10). Owing to the small group sizes (n=3 per condition), between-group differences were not tested. However, these overall scores suggested the potential for ceiling effects in the item’s responses. Items were revised slightly to minimize these effects, but attention should be paid to this during the trial.

The recall instrument required revision (NL and ES), as some items appeared overly easy or complex. Adaptations resulted in a total pool of 28 items (14 active recall and 14 recognition). For active recall, possible scores now range from 0 to 33 for recognition from 0 to 14. The pilot test indicated sufficient variation for information satisfaction, physician trust, and expected symptom distress.

Taken together, the results of the pilot test and subsequent revisions support the start of the trial. The procedures of the planned trial are further detailed below.

### Participant Timeline

Participant recruitment and data collection are expected to last up to 2 months to reach the required sample size. This is considered feasible, based on previous experience using participant panels for study recruitment.

### Sample Size

The required sample size is estimated at N=420 participants (*structuring* N=180 and *tailoring* N=240), based on *a priori* power analyses in G*Power [75] with the alpha set at .05, a probability level of .80, and estimated medium effect sizes of .10 to .25 for the dependent variables, that is, information recall, information satisfaction, and trust in the physician.

### Recruitment Procedures

First, members of the PanelCom panel will be invited to participate in the experiment via mass emailing, receiving up to 2 reminders [65]. Second, (former) patients with cancer and their family members will be recruited in collaboration with several cancer patient support organizations, including the Dutch Cancer Society and Hematon, the Dutch association for patients with blood cancer and lymphoma. Through these organizations, potential participants will be informed about the study and invited to sign up for participation.

Participants are informed that the study is part of a research project about information giving in the context of cancer treatment. Furthermore, they are informed that study participation includes an online survey and a scripted video of a hematology consultation that will take approximately 30 min to complete and can be entered from a home computer. Participants are asked to complete the survey individually and in one sitting. Finally, participants will be notified that all data will be treated confidentially and remain anonymous. Participants provide informed consent upon entering the online survey.

### Allocation and Blinding

Participants are automatically and randomly assigned to 1 of 12 conditions, or interventions, (1:1 ratio) in either the structuring or the tailoring experiment. Allocation is achieved by computer-generated randomization. Participants are unaware to which condition, that is, intervention, they are assigned (blinded). Researchers are unaware, for the duration of the trial, to which condition participants are assigned.

### Analysis

Data will be cleaned in a stepwise procedure [76], including the identification of missing values because of dropout, standardization and normalization of data, and outlier analysis. Data will subsequently be analyzed using IBM SPSS statistical package. In the first step, it will be determined whether the experimental groups, within each of the 2 subexperiments of the trial, are indeed comparable in terms of participant characteristics, such as sociodemographics, personality traits, and disease history. If differences are found, these will be controlled for in subsequent analyses. Then, for each of the 2 experiments, one-way analysis of variance or covariance (when aspects need to be controlled for) will be conducted to test the effects of information structuring, information tailoring, and caring on information recall, satisfaction, and trust (H1, H2, and H3, respectively). Additionally, the interaction effect between caring and information structuring as well as tailoring will be added to these models (RQ1). It will be investigated to what extent patient characteristics moderate the hypothesized relationships (RQ3). If necessary, because of violations of the assumption of homogeneity of variance, Welch F statistic will be employed. Posthoc comparisons, using Bonferroni or Games-Howell, will be used as applicable, to create a better understanding of between-group differences. Finally, linear regression analyses will be used to assess possible relationships of information recall, satisfaction, and trust with expected symptom distress (RQ2). Significance levels are determined at *P*<.05.

## Results

Data collection has now been completed. A total of N=607 participants went to our homepage, provided informed consent online, and started the survey. A total of N=470 completed the first part of the survey and were randomized to receive 1 of the 12 video vignette interventions within 1 of the 2 experiments (77.4%, 470/607). Participants did not differ from those who dropped out, except for their age (in experiment 1, N=148): completers were younger (mean 3.8 years; *P*=.002) and consequently less likely to be retired (41.2%, 194/470) vs 56.4% (83/148; *P*=.006)*.* The first analyses will be available in Spring 2019.

## Discussion

### Strengths and Limitations

This study protocol describes the procedures for a randomized controlled trial in which 2 video vignette experiments are used to test the effects of physician information giving about side effects of cancer treatment on patient outcomes. Specifically, the effects of cognitive-oriented communication strategies (ie, information structuring and tailoring) as well as affect-oriented strategies (ie, caring) on patient recall, satisfaction, and trust are tested in conjunction. The outlined approach has both advantages and limitations.

Video vignette experiments allow researchers to experimentally test the causal relationships between communicative behaviors and consultation outcomes. This is particularly relevant when systematic manipulation of physicians’ behaviors is undesirable for ethical or practical reasons. This trial thus has the potential to yield critical evidence to support interventions to change communicative behaviors in clinical practice. In the preparatory phase of the trial, the video vignettes were carefully developed in a stepwise procedure, involving a panel of hematologists, patients with cancer, health communication researchers, and medical psychologists as well as a pilot test. Through this procedure, the script and its manipulations were thoroughly evaluated to ensure vignette realism as well as the effectiveness of the separate manipulations.

However, it should be noted that design artificiality can hamper ecological validity and that the use of analogue patients can hinder participants’ ability to identify with the portrayed clinical situation. To ensure vignette realism, the script was based on a transcript of a full-length hematology consultation. As such, the script sought to represent a true-to-life hematology consultation rather than an ideal situation. Duration differences between the different versions of vignettes might account for differences in outcomes rather than the manipulation. However, duration differences are characteristic for realistic consultations, and compensating for these differences by adding *fillers* to the script may produce its own, undesirable, effects [63].

The pilot study demonstrated that participants were indeed able to identify and engage with the video patient. Inclusion of study participants who have previous experience with oncology consultations is expected to further improve identification with the vignettes, although our research group previously found no difference in identification between patients with cancer and cancer naïve participants [63].

As participants will be recruited via a panel of (former) patients with cancer and their relatives, as well as via patient organizations, it should be taken into account that panel participants may not be fully representative of the patient population. However, this is not deemed problematic as we primarily aim to identify pathways underlying effective information giving rather than to generalize patient outcomes to the population. The use of participants with previous experience with cancer does raise ethical concerns, as participants may experience feelings of anxiousness or sadness as a result of viewing a video in which a cancer treatment plan is discussed. This was reviewed by the institutional ethics committee. Participants are extensively debriefed following the experiment to minimize any negative impact of study participation.

Finally, it should be noted that, although video vignettes provide an effective method to study communication effects among (oncology) patients and their relatives, sometimes the use of so-called analogue patients poses a challenge. When striving to test the effects of information tailoring, the communication in the scripted vignettes is tailored to the video-patient rather than to the study participant. This may have implications for the findings. Direct effects of tailoring on participants’ recall of information cannot be assumed. To overcome this issue, we added an item to the survey, assessing participants’ personal information preferences (amount). Consequently, we can control for this variable in our statistical models.

### Implications

The results of the proposed trial will provide evidence concerning the pathways linking physician communication to (improved) consultation outcomes for patients. In particular, the relationships between physicians’ information structuring and tailoring (cognition-oriented skills) and caring (affect-oriented skill) and patients’ recall (cognitive outcome), satisfaction, trust in the physician, and—ultimately—symptom distress (affective outcomes) will be clarified. The trial will allow researchers to further define what effective information provision about treatment precisely entails. Thereby, this study is highly relevant for patient-provider research in oncology settings. However, there are also practical implications. The results can be used to improve medical education about information provision. Within the scope of this study, it is indeed aimed to develop an innovative, evidence-based training module for hematologists about treatment information provision in cancer care [77]. The ultimate aim of this study is to contribute to our understanding of how oncologists can best inform patients about future symptoms to eventually improve patients’ well-being and minimize potential suffering.

## Abbreviations

DLBCL: diffuse large B-cell lymphoma
